# Pathogenic *Escherichia coli* isolated from recreational waters in northwestern Wyoming display multidrug resistance

**DOI:** 10.1093/jambio/lxag240

**Published:** 2026-09-21

**Authors:** Anahita Ghorbani Tajani, Aniket Sharma, Kelsey C Ruehling, Bledar Bisha

**Affiliations:** Department of Animal Science, University of Wyoming, Laramie, WY 82071, United States; Department of Animal Science, University of Wyoming, Laramie, WY 82071, United States; Department of Zoology and Physiology, University of Wyoming, Laramie, WY 82071, United States; Department of Animal Science, University of Wyoming, Laramie, WY 82071, United States

**Keywords:** antimicrobial resistance, *Escherichia coli*, multidrug-resistant bacteria, plasmids, environmental/recreational water

## Abstract

**Aim:**

To determine the prevalence, antimicrobial resistance, and genomic characteristics of antimicrobial-resistant *Escherichia coli* isolated from recreational freshwater in the Greys-Hoback watershed, Wyoming, USA.

**Methods and results:**

A total of 263 presumptive *E. coli* isolates were recovered using membrane filtration (EPA Method 1603), of which 218 (83%) were confirmed as *E. coli* by matrix-assisted laser desorption/ionization time-of-flight mass spectrometry (MALDI-TOF MS). Antimicrobial susceptibility testing by broth microdilution identified 20 (∼9%) isolates with clinically relevant resistance phenotypes, including six multidrug-resistant (MDR; ≥3 antimicrobial classes). Whole-genome sequencing of the resistant subset identified *bla*_EC_ alleles in all genomes, along with additional resistance determinants, including *sul*2, *tet, aph* variants, and *floR* genes. Plasmid replicons were detected in most isolates, with IncF-type plasmids predominating and associated with MDR gene clusters. Contig-level analysis identified plasmid-associated MDR regions in selected isolates, although plasmid reconstruction was limited by short-read assemblies. Phylogenetic analyses revealed multiple sequence types and O: H serotypes, demonstrating substantial genetic diversity and suggesting that the resistant *E. coli* population originated from multiple sources rather than from clonal expansion. All sequenced isolates carried multiple virulence-associated genes, indicating co-occurrence of resistance and virulence determinants.

**Conclusions:**

By integrating antimicrobial susceptibility testing, whole-genome sequencing, and plasmid reconstruction, we show that recreational freshwater in northwestern Wyoming harbors genetically diverse antimicrobial-resistant pathogenic *E. coli*, including MDR isolates associated with putative mobile resistance elements. These findings support the inclusion of recreational water systems in environmental AMR surveillance frameworks and highlight the value of integrating phenotypic and genomic approaches for isolate characterization.

Impact Statement Most antimicrobial resistance (AMR) surveillance focuses on clinical, agricultural, and wastewater settings, often overlooking environmental settings where people may interact with contaminated water. Recreational rivers are infrequently considered in AMR policy, yet they are places where humans, livestock, wildlife, and antibiotic residues intersect, making them crucial “zones” within the AMR network. The present study demonstrates a practical approach to transforming these sites into AMR indicators by combining standard water quality monitoring with whole-genome sequencing, providing an actionable framework for earlier detection of high-risk bacterial lineages, more informed recreation advisories, and targeted upstream interventions within a One Health framework.

## Introduction

Antimicrobials have extended human life expectancy by at least 20 years by enabling effective treatment of bacterial infections. However, widespread antibiotic use has accelerated the emergence of antimicrobial resistance (AMR). The World Health Organization identifies AMR as one of the top ten global public health threats and projects that it will contribute substantially to global mortality and economic losses by 2050. These resistant bacteria are ubiquitous across diverse environments, including meat, wastewater, freshwater, and other environmental reservoirs (Antimicrobial Resistance Collaborators 2022, Boidya *et al*. 2025, Ghorbani Tajani *et al*. 2026, Kaur *et al*. 2026).

In addition to its impact on human health, AMR also imposes substantial economic challenges, increases healthcare costs, extends hospital stays, and reduces productivity (Kraemer *et al*. 2022). Antimicrobial resistance genes (ARGs) have been present in natural ecosystems for millennia as intrinsic defense mechanisms against naturally occurring antimicrobials present in plants and other environmental stressors (Perry *et al*. 2014). Their increasing detection in clinically important pathogens is particularly concerning, especially in regions with inadequate water, sanitation, and hygiene infrastructure. A 2015 survey in the Netherlands found that 17.1% of Extended-Spectrum Beta-Lactamases (ESBL)-producing *E. coli* from river water and wastewater were classified as pathogenic based on virulence profiling, and among these pathogenic isolates, ∼84% were resistant to up to three antibiotic classes (Franz *et al*. 2015). In 2019, a study from northern Colorado showed that, although total bacteria and *E. coli* abundances decreased through wastewater treatment, the relative proportion of ESBL-resistant *E. coli* increased by 1.7% in surface waters, with 70 isolates validated across 17 antibiotics and 185 AMR genes detected via whole-genome sequencing (WGS; Haberecht *et al*. 2019). Additionally, in Wyoming, *Bacillus cereus* strain IM8, isolated from an Upper Fremont Glacier ice core, displayed high cytotoxicity toward human intestinal epithelial Caco-2 cells and resistance to ampicillin (Chandross-Cohen et al. 2026). Together, these findings highlight the importance of monitoring environmental bacteria for AMR and virulence traits across diverse water sources and geographic regions.

Exposure through recreational activities, such as swimming, fishing, or agricultural irrigation, can lead to ingestion or contact, resulting in infections or colonization by resistant bacteria. In arid and semi-arid regions like the northwestern USA, AMR risks are amplified by agricultural activity and limited sewage treatment infrastructure (Kaur *et al*. 2026). Wyoming, with its extensive ranching operations and decentralized waste management, faces unique water quality challenges. Recent assessments of Fish and Flat Creeks near Jackson, a gateway to the US’s oldest national park and a popular tourist spot, have reported *E. coli* levels exceeding recreational water standards, raising concerns about waterborne transmission of AMR (Wyoming Department of Environmental Quality 2017).

Traditionally, fecal indicator bacteria such as *E. coli* and *Enterococcus* spp. have been used to assess water quality through culture-based methods. However, these approaches provide limited insight into microbial community structure, resistance mechanisms, and genetic exchange among bacteria (Korajkic *et al*. 2018). Pairing culture-based approaches with advanced molecular techniques, including next-generation sequencing and metagenomics, provides powerful tools for characterizing microbial diversity, ARG prevalence, and resistance-transmission pathways (Sukhum *et al*. 2019). We hypothesized that recreational waters may serve as reservoirs of mobile MDR gene clusters in *E. coli*, reflecting diverse anthropogenic and zoonotic inputs.

Despite growing attention to AMR in clinical, agricultural, and wastewater settings, impaired recreational watersheds remain rarely characterized at the genomic level, and few studies have integrated phenotypic susceptibility testing, whole-genome sequencing, and plasmid reconstruction to resolve where resistance determinants reside and how mobile they are. To our knowledge, this is among the first studies to apply this combined approach to an impaired recreational freshwater system. Therefore, the objectives of this study were to isolate *E. coli* from freshwater samples and characterize their phenotypic resistance profiles, genomic resistance determinants, and clonal relatedness across sampling sites. We further hypothesized that isolates from this impaired recreational watershed would include genetically diverse lineages carrying ExPEC-like virulence traits and MDR plasmids.

## Materials and methods

### Study area and sampling locations

Presumptive *E. coli* isolates (*n* = 263) were collected in 2021 from the Greys-Hoback watershed in Wyoming. Fourteen sampling sites were chosen along Fish and Flat Creeks, both designated as impaired due to elevated *E. coli* levels, as well as their tributaries, Cache and Game Creeks. Sites were selected to represent upstream, midstream, and downstream locations across the watershed while maintaining accessibility for consistent sampling.

### Sample collection and isolate selection

Surface water samples were collected ∼15 cm below the water surface using sterile 1 l polypropylene bottles and a sampling pole to minimize substrate disturbance. Samples were transported on ice and processed within 24 h, filtered through 0.45 µm membrane filters (Pall Corporation, USA) to capture bacteria. The filtered material was then cultured on commercially available modified membrane-thermotolerant *E. coli* agar (modified mTEC) (Sigma-Aldrich, USA) for selection of *E. coli*. For quality control, *E. coli* (ATCC 11775) was used as a positive control, and *Enterococcus faecalis* (ATCC 19433) as a negative control. Isolates were confirmed *via* MALDI-TOF MS (Bruker Microflex LRF, Biotyper version 3.0, USA), using a bacterial test standard (Bruker, USA) as a control to identify the colonies. Fresh, isolated colonies were placed on a target plate, dried, and coated with α-cyano-4-hydroxycinnamic acid matrix (Bruker, USA). Scores ≥ 2.300 indicate species-level identification (Popović *et al*. 2022).

### Antimicrobial susceptibility testing

Antimicrobial susceptibility testing (AST) was assessed by broth microdilution according to Clinical and Laboratory Standards Institute (CLSI) guidelines (M100-ED35:2025) using the Sensititre system (Thermo Fisher Scientific, USA). In brief, well-isolated overnight colonies were suspended in sterile 0.85% saline and adjusted to a 0.5 McFarland standard using a nephelometer (CrystalSpec). This suspension was then diluted in Mueller-Hinton broth (Thermo Fisher Scientific) to achieve a final inoculum density of approximately 5 × 10^5^ CFU ml^−1^ in accordance with CLSI guidelines.

Pre-coated antibiotic plates (CMV3GANF, Thermo Fisher Scientific, USA) were inoculated with 50 μl of suspension and incubated at 37°C for 18–20 h. Minimum inhibitory concentrations (MICs) were determined using SWIN software (v3.3) (Thermo Fisher Scientific, 2024, USA) and interpreted in accordance with CLSI standards (EM100 Connect—CLSI M100 ED35:2025,n.d.). The antimicrobials tested and their respective concentration ranges (µg ml^−1^) were gentamicin (0.25–16), streptomycin (2–64), amoxicillin-clavulanic acid (1/0.5–32/16), ceftriaxone (0.25–64), ceftiofur (0.12–8), cefoxitin (0.5–32), sulfisoxazole (16–256), trimethoprim-sulfamethoxazole (0.12/2.38–4/76), azithromycin (0.12–16), ampicillin (1–32), chloramphenicol (2–32), ciprofloxacin (0.015–4), nalidixic acid (0.5–32), and tetracycline (4–32).

### DNA Extraction and Library Preparation

*Escherichia coli* isolates exhibiting phenotypic resistance to at least one of the tested antimicrobials were selected for WGS, as described previously (Ghorbani Tajani *et al*. 2024). In brief, cultures were grown on brain heart infusion agar (Oxoid, USA) at 37°C for ∼18 h. DNA extraction was performed using the DNeasy Blood & Tissue Kit on a QIAcube system (Qiagen) at the Wyoming Public Health Laboratory (Cheyenne, Wyoming), following the manufacturer’s instructions. Libraries were prepared using the NEBNext Ultra DNA Library Prep Kit (NEB, USA). DNA was sonicated to an average fragment size of 350 bp, end-repaired, A-tailed, adapter-ligated, and PCR-amplified with five cycles of indexing. Library quality was assessed using an Agilent 2100 Bioanalyzer, and DNA concentration was measured with a Qubit 2.0 Fluorometer (Thermo Fisher Scientific, USA).

### Whole Genome Sequencing and Bioinformatics

Sequencing was performed on an Illumina NovaSeq 6000 platform using 2 × 150-cycle paired-end reads. Raw sequencing reads were evaluated as described previously (Ghorbani Tajani et al. 2025). Briefly, reads were assessed using FastQC (v0.12.0), and low-quality bases and adapters were trimmed using Trimmomatic (v0.36). High-quality reads were assembled *de novo* using SPAdes (v3.15.5), and genome annotation was performed using the NCBI Prokaryotic Genome Annotation Pipeline (PGAP; v6.8).

ARGs were identified using ABRicate (v1.0.1), and plasmid replicons using PlasmidFinder (v2.0.1). Multilocus Sequence Typing (MLST) *via* MLST software (v2.0.9), and core genome MLST (cgMLST) analysis conducted with cgMLSTFinder (v1.2). Virulence factors and pathogenic potential were assessed using VirulenceFinder (v2.0) and PathogenFinder (v1.1), respectively. Additional resistance gene profiling was performed using ResFinder (v4.6.0) with the Center for Genomic Epidemiology (CGE) Database (https://www.genomicepidemiology.org/services/). The phylogenetic relationships along with *fimH* typing were inferred using EnteroBase (v.1.2.0) (https://enterobase.warwick.ac.uk/). Phylogroup assignments were also obtained through EnteroBase and incorporated into the phylogenetic tree to characterize the broader phylogenetic distribution of the isolates.

Draft genome assemblies were screened using MobileElementFinder (v1.0.3) (using the CGE server) to identify mobile genetic elements at the contig level. Based on this analysis, three isolates (S5, S7, and S30) were selected because they showed co-occurrence of plasmid replicons and AMR genes on the same contigs.

Selected assemblies were further analyzed using MOB-suite (mob_recon and mob_typer; v3.1.9) to assign contigs to putative plasmid clusters and predict plasmid mobility based on replicon content, relaxase type, mating pair formation (MPF) system, and oriT-associated features. AMR genes were further identified by screening assemblies and plasmid-associated contigs against the NCBI AMR database using ABRicate (v1.0.1). Resistance genes were considered plasmid-associated only when located on contigs classified as plasmid-derived by MOB-suite. Because plasmid reconstruction from short-read assemblies can be affected by fragmentation and repetitive sequences, results were interpreted conservatively, and ambiguous contigs were not classified as definitively plasmid-borne.

### Data Analysis

Statistical and bioinformatic analyses were conducted to characterize AMR, plasmid content, and virulence gene profiles among *E. coli* isolates. Genomic data were structured as binary presence/absence matrices for ARGs, plasmid replicons, and virulence-associated genes using Python (v3.12) and the pandas library. Prevalence was calculated as the proportion of isolates carrying each genetic feature.

Comparisons of gene prevalence were performed using Fisher’s exact test, appropriate for small sample sizes and categorical data. To account for multiple comparisons, the Bonferroni correction was applied, and adjusted *P-values* < 0.05 were considered statistically significant.

All statistical analyses were conducted on the subset of phenotypically resistant isolates selected for genomic characterization and therefore do not represent the full *E. coli* population recovered from the watershed.

## Results

### Multidrug-resistant *E. coli* prevalence in a recreational freshwater system

A total of 218 isolates were confirmed as *E. coli* by MALDI-TOF MS (Bruker, USA), providing the pool from which antibiotic-resistant isolates were selected. AST identified 20 (∼9%) isolates with clinically relevant resistance profiles (Isolates exhibiting resistance to at least one antimicrobial were considered AMR). The resistant isolates were recovered from multiple sampling locations across the watershed, indicating a broad spatial distribution rather than restriction to a single sampling location. Additionally, six isolates (S5, S6, S24, S26, S29, and S30) were found to be MDR, defined as resistance to three or more antimicrobial classes (Fig. 1). Sampling dates for the 20 phenotypically characterized isolates spanned the 2021 recreation season, from early spring (March) through late September.

**Figure 1:**
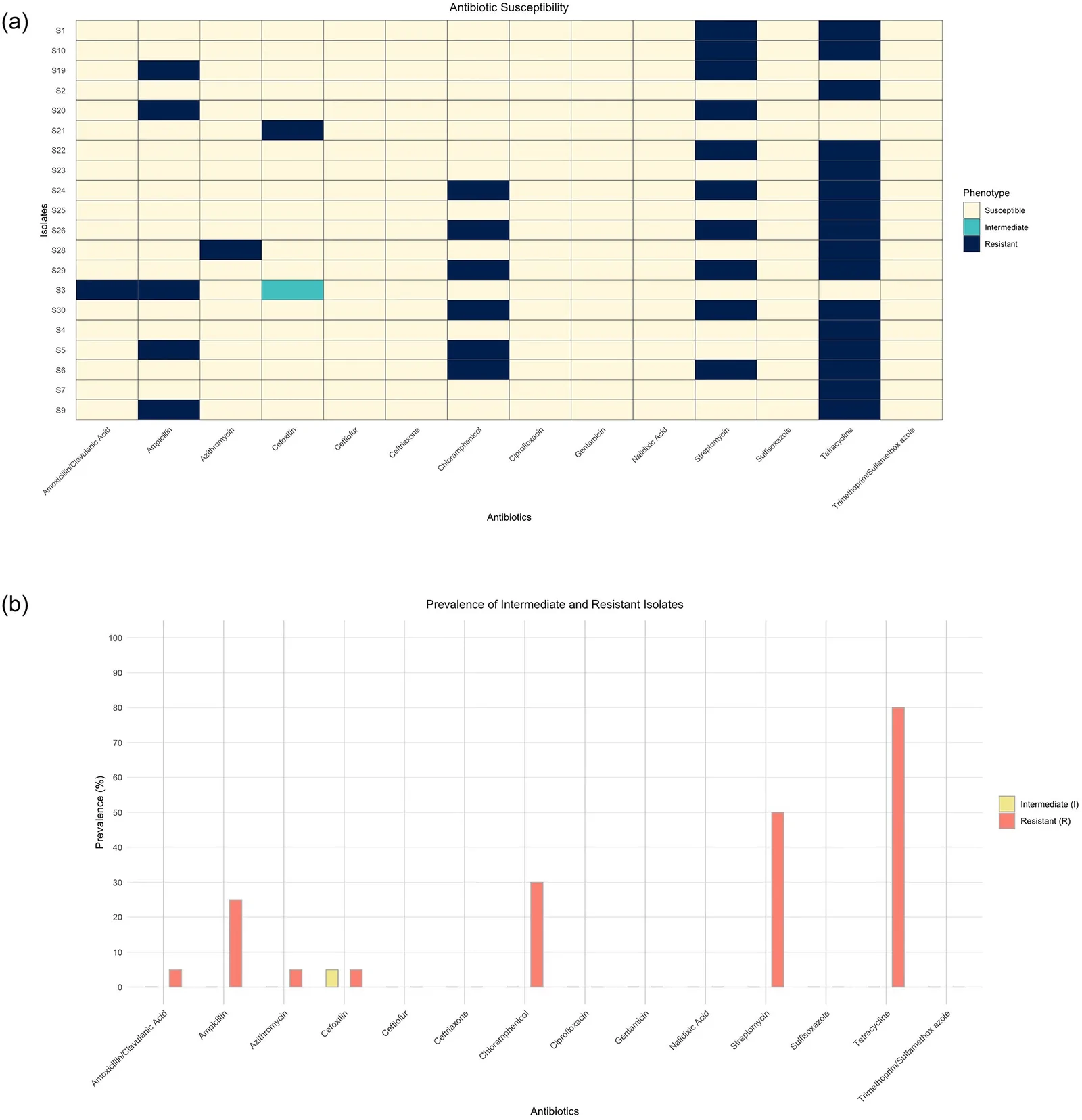
(a) Antibiotic susceptibility profiles of *E. coli* isolates (*n* = 20) collected from freshwater in the Greys-Hoback watershed. The heatmap shows the susceptibility of individual isolates (y-axis) to a panel of 14 antibiotics (x-axis), categorized as susceptible, intermediate, or resistant according to established clinical breakpoints. Visualization was generated in R (v4.5.2). (b) Prevalence (%) of intermediate and resistant isolates across tested antimicrobials. Resistance (R) and intermediate (I) were determined based on AST results for *E. coli* isolates (*n* = 20). Visualization was generated in R (v4.5.2).

### Antibiotic resistance patterns in *E. coli* isolates

Among these 20 resistant isolates, tetracycline resistance was most frequent (16/20, 80%), followed by streptomycin (10/20, 50%). Interestingly, isolate S21 was the only *E. coli* found to be resistant to cefoxitin, while isolate S3 displayed intermediate resistance to this second-generation cephalosporin. All isolates remained susceptible to third-generation cephalosporins and several other clinically important agents (Fig. 1), indicating that resistance was largely confined to older, commonly used antibiotic classes.

### Genomic characterization of MDR and virulence in freshwater *E. coli*

Genomic and statistical analyses were restricted to the 20 phenotypically resistant *E. coli* isolates selected for WGS. The *bla*_EC_ gene family was detected in all 20 genomes (100%). The *bla*_EC-18_ allele was the most common (13/20, 65%) and was significantly more prevalent than other detected *bla*_EC_ alleles after Bonferroni correction (adjusted *P* < 0.05, Table 1, Fig. 2a).

**Figure 2:**
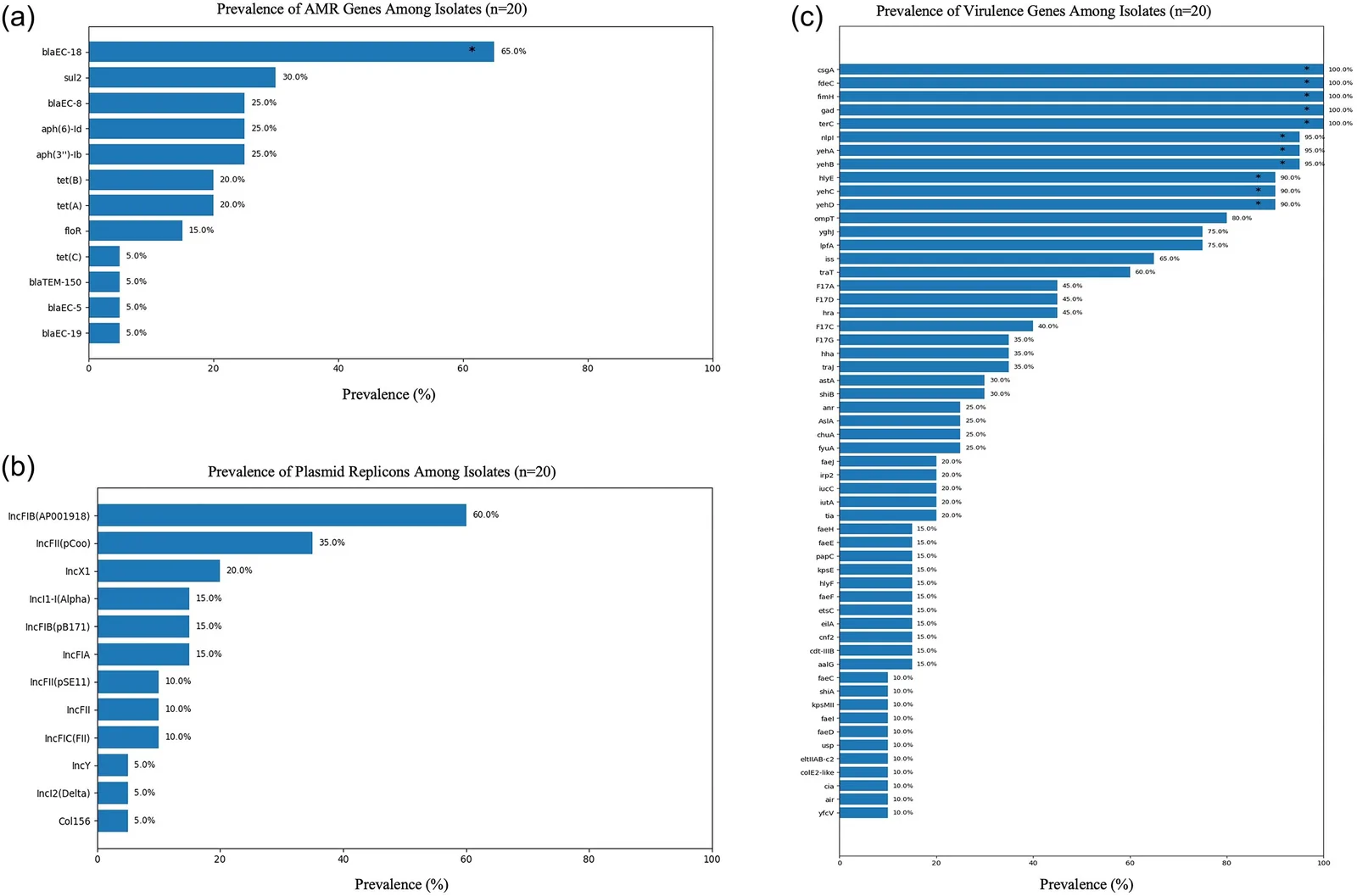
Prevalence of (a) Antimicrobial Resistance Genes, (b) Plasmid Replicons, and (c) Virulence Genes among *E. coli* isolates (*n* = 20). Prevalence was calculated as the percentage of isolates in which each marker was detected based on whole-genome sequencing data. For each isolate, marker presence was treated as a binary variable (present/absent), and duplicate entries within isolates were removed. Plasmid entries reported as “No hits found” (*n* = 3) were excluded from analysis. Visualization was generated using Matplotlib in Python (v3.12).

**Table 1:** Genomic characteristics of antibiotic-resistant *E. coli* isolates (*n* = 20) recovered from freshwater samples of the Greys-Hoback watershed.

Isolates	AMR Gene(s)	Plasmid(s)	Virulence Genes	Pathogenicity (%)	GPS Coordinates	Accession Number
S1	*bla*_EC-19_	IncFIB(AP001918), IncFII	*AslA, aalG, anr, astA, chuA, csgA, faeC, faeD, faeE, faeF, faeH, fdeC, fimH, fyuA, gad, irp2, kpsE, kpsMII, nlpI, ompT, papA_F1651A, papC, shiA, terC, traT, usp, yehA, yehB, yehC, yehD, yfcV, yghJ*	Yes (93.7%)	43.393935°, −110.727465°	JBIEAL000000000
S2	*bla*_EC-18_, *tet(C)*	IncFIB(AP001918), IncFII(pSE11), IncI1-I(Alpha), IncX1	*AslA, F17A, F17C, F17D, F17G, cdt-IIIB, chuA, cnf2, csgA, eilA, eltIIAB-c1, espY2, faeC, faeD, faeE, faeF, faeH, faeI, faeJ, fdeC, fimH, gad, hlyE, iss, iucC, iutA, nlpI, ompT, terC, traT, yehB*	Yes (92.9%)	43.460454°, −110.864262°	JBIEAK000000000
S3	*bla*_EC-18_	IncFIB(pB171), IncFII(pCoo)	*csgA, fdeC, fimH, gad, hlyE, lpfA, nlpI, ompT, terC, traJ, traT, yehA, yehB, yehC, yehD, yghJ*	Yes (94.6%)	43.523742°, −110.863134°	JBIEAJ000000000
S4	*bla*_EC-18_, *tet(B)*	IncFIA, IncFIB(AP001918), IncFIB(pB171), IncFII(pCoo)	*astA, csgA, fdeC, fimH, gad, hlyE, hra, iss, lpfA, nlpI, ompT, shiB, terC, traT, yehA, yehB, yehC, yehD*	Yes (93.5%)	43.460454°, −110.864262°	JBIEAI000000000
S5	*bla*_EC-18_ *, floR, tet(A), aph(6)-Id, aph(3′’)-Ib, sul2, bla*_TEM-150_	IncFIA, IncFIB(AP001918), IncFIC(FII)	*anr, csgA, fdeC, fimH, gad, hlyE, iss, lpfA, nlpI, ompT, terC, traJ, traT, yehA, yehB, yehC, yehD*	Yes (94.1%)	43.49565°, −110.871933°	JBIEAH000000000
S6	*bla*_EC-18_	No hits found	*F17A, F17C, F17D, F17G, astA, csgA, fdeC, fimH, gad, hlyE, hra, iss, lpfA, nlpI, ompT, shiB, terC, yehA, yehB, yehC, yehD, yghJ*	Yes (93.7%)	43.39056°, −110.748393°	JBIEAG000000000
S7	*tet(A), bla*_EC-18_	IncFIB(AP001918), IncFII(pCoo), IncI1-I(Alpha)	*csgA, fdeC, fimH, gad, hlyE, lpfA, nlpI, ompT, terC, yehA, yehB, yehC, yehD, yghJ*	Yes (94.3%)	43.49565°, −110.871933°	JBIDZM000000000
S9	*bla*_EC-18_, *tet(B)*	IncFIB(AP001918), IncY	*csgA, fdeC, fimH, gad, hlyE, lpfA, nlpI, ompT, terC, traT, yehA, yehB, yehC, yehD, yghJ*	Yes (94.0%)	43.48778°, −110.763211°	JBIEAD000000000
S10	*aph(3′’)-Ib, aph(6)-Id, sul2, bla*_EC-18_ *, tet(B)*	IncFIB(AP001918), IncFII(pCoo)	*aamR, csgA, fdeC, fimH, gad, hlyE, hra, iss, lpfA, nlpI, ompT, papC, shiA, shiB, terC, tia, traT, yehA, yehB, yehC, yehD, yghJ*	Yes (93.8%)	43.523742°, −110.863134°	JBIEAC000000000
S19	*bla*_EC-18_	IncFII, IncX1	*AslA, F17A, F17C, F17D, air, astA, chuA, csgA, eilA, fdeC, fimH, gad, hha, hlyE, hra, iss, nlpI, shiB, terC, tia, traT, yehA*	Yes (92.8%)	43.460454°, −110.864262°	JBIDZU000000000
S20	*bla*_EC-18_	IncI2(Delta)	*colE2-like, csgA, fdeC, fimH, gad, hlyE, iss, lpfA, nlpI, ompT, terC, yehA, yehB, yehC, yehD, yghJ*	Yes (94.4%)	43.39056°, −110.748393°	JBIDZT000000000
S21	*bla*_EC-18_ *, sul2*	IncX1	*F17A, F17C, F17D, csgA, fdeC, fimH, gad, hha, hlyE, hra, nlpI, shiB, terC, yehA, yehB, yehC, yehD, yghJ*	Yes (93.5%)	43.39056°, −110.748393°	JBIDZS000000000
S22	*bla*_EC-18_ *, sul2, aph(6)-Id, aph(3′’)-Ib, tet(B)*	IncFIB(pB171), IncFII(pCoo)	*F17A, F17D, F17G, astA, csgA, fdeC, fimH, fyuA, gad, hha, hlyE, hra, irp2, lpfA, ompT, terC, tia, traJ, traT, yehA, yehB, yehC, yehD, yghJ*	Yes (93.6%)	43.45835°, −110.797119°	JBIDZR000000000
S23	*bla*_EC-5_ *, tet(A)*	IncFIA, IncFIB(AP001918), IncFIC(FII)	*AslA, anr, chuA, csgA, cvaC, hak, etsC, fdeC, fimH, fyuA, gad, hlyF, ibeA, iroN, irp2, iss, iucC, iutA, kpsE, kpsMII_K1, mchF, neuC, nlpI, ompT, sitA, terC, traJ, traT, tsh, usp, vat, yehA, yehB, yehC, yehD, yfcV*	Yes (93.9%)	43.39056°, −110.748393°	JBIDZQ000000000
S24	*bla*_EC-18_ *, sul2, aph(3′’)-Ib, aph(6)-Id, tet(A), floR*	IncFIB(AP001918), IncFII(pCoo)	*F17A, F17C, F17D, F17G, csgA, eltIIAB-c3, fdeC, fimH, fyuA, gad, hha, hlyE, hra, irp2, iss, lpfA, nlpI, shiB, terC, traJ, yehA, yehB, yehC, yehD, yghJ*	Yes (93.9%)	43.523742°, −110.863134°	JBIDZP000000000
S25	*bla*_EC-8_	Col156, IncFIB(AP001918), IncI1-I(Alpha), IncX1	*AslA, air, chuA, colE2-like, csgA, eilA, faeE, faeF, faeH, faeI, faeJ, fdeC, fimH, gad, hha, hlyE, iss, kpsE, kpsMII, lpfA, nlpI, ompT, papC, terC, yehA, yehB, yehC, yehD, yghJ*	Yes (93.6%)	43.574643°, −110.836912°	JBIDZO000000000
S26	*bla*_EC-8_	No hits found	*csgA, fdeC, fimH, gad, hha, hlyE, hra, lpfA, nlpI, terC, yehA, yehB, yehC, yehD, yghJ*	Yes (94.3%)	43.432652°, −110.792589°	JBIDZN000000000
S28	*bla*_EC-8_	No hits found	*F17A, F17C, F17D, F17G, astA, csgA, fdeC, fimH, fyuA, gad, hha, hlyE, hra, iss, lpfA, nlpI, ompT, terC, tia, yehA, yehB, yehC, yehD, yghJ*	Yes (93.8%)	43.432652°, −110.792589°	JBIDZL000000000
S29	*bla*_EC-8_	IncFIB(AP001918), IncFII(pSE11)	*F17A, F17C, F17D, F17G, aalG, anr, cdt-IIIB, cia, cnf2, csgA, eltIIAB-c2, etsC, faeJ, fdeC, fimH, gad, hlyE, hlyF, iss, iucC, iutA, lpfA, nlpI, ompT, terC, traJ, traT, yehA, yehB, yehC, yehD, yghJ*	Yes (94.1%)	43.39056°, −110.748393°	JBIDZK000000000
S30	*bla*_EC-8_ *, floR, tet(A), aph(6)-Id, aph(3′')-Ib, sul2*	IncFIB(AP001918), IncFII(pCoo)	*F17A, F17C, F17D, F17G, aalG, anr, cdt-IIIB, cia, cnf2, csgA, eltIIAB-c2, etsC, faeJ, fdeC, fimH, gad, hlyE, hlyF, iss, iucC, iutA, lpfA, nlpI, ompT, terC, traJ, traT, yehA, yehB, yehC, yehD, yghJ*	Yes (93.9%)	43.523742°, −110.863134°	JBIDZJ000000000

Less common β-lactam determinants included *bla*_TEM-150_, which was detected only in isolate S5, as well as additional *bla*_EC_ variants such as *bla*_EC-19_, *bla*_EC-5_, and *bla*_EC-8_. Non-β-lactam ARGs were more frequently detected, including tetracycline resistance genes (*tet*(A)*, tet*(B), *tet*(C)) (10/20, 50%), the sulfonamide resistance gene *sul2* (6/20, 30%), aminoglycoside resistance genes *aph(3′')-Ib* and *aph(6)-Id* (each 5/20, 25%), and the phenicol resistance gene *floR* (3/20, 15%) (Table 1, Fig. 2a).

Plasmid replicons were detected in 17 of the 20 (85%) *E. coli* isolates, with many isolates harboring multiple incompatibility groups. Isolates S6, S26, and S28 lacked any detectable plasmid replicons, whereas isolates S2 and S25 both carried the greatest diversity of plasmid types, each harboring four replicon families (IncFIB(AP001918), IncFII[pSE11 or pCoo], IncI1-I(Alpha), and IncX1 or Col156).

IncF-type replicons predominated with IncFIB(AP001918) detected in 12/20 isolates (60%) and IncFII variants (including IncFII(pCoo) and IncFII(pSE11)) also present in 11/20 isolates (55%). Additional plasmid backbones included IncX1 and IncFIA (4/20, 20%; 3/20, 15% respectively), with IncI1-I(Alpha) (3/20, 15%), IncI2(Delta) (1/20, 5%), IncY (1/20, 5%), and the small Col156 replicon (1/20, 5%). These findings indicate that multi-replicon IncF plasmids constitute the dominant vehicles for ARG carriage in this *E. coli* population (Table 1, Fig. 2b). Notably, S5 combined the highest ARG count with three IncF plasmids (Table 1, Fig. 2b), highlighting this isolate as a notable MDR, plasmid-rich genotype.

All *E. coli* isolates harbored multiple virulence-associated genes (20/20, 100%) (Table 1, Fig. 2c). Most isolates (including S3-S7, S9, S10, S19-S22, S24-S26, S28-S30) carried core adherence and serum-resistance factors such as *csgA, fdeC, fimH, gad, hlyE, lpfA, nlpI, ompT, terC*, and the tellurite resistance locus *yehA-yehD*. Several isolates (S1, S19, S21, S22, S23, S24, S25, S28–S30) showed expanded ExPEC- and ETEC-associated repertoires, encoding combinations of *fyuA* and/or *irp2* (yersiniabactin siderophore system), *kpsE*/*kpsMII* (capsule), *papA/papC* (P fimbriae), *eltIIAB* variants (LT toxin), *tsh, vat, ibeA, iroN*, and multiple *F17* fimbrial variants (*F17A, F17C, F17D, F17G*). Toxin and intestinal pathogenicity markers such as *astA, air, cvaC, colE2-*like, *cdt-IIIB, elt-IIAB, eilA*, and *hlyF* were detected in subsets of isolates (notably S2, S19, S22-S25, S29, and S30), indicating that several strains combine extraintestinal and intestinal virulence traits. Eleven virulence genes (*csgA, fdeC, fimH, gad, terC, nlpI, yehA, yehB, hlyE, yehC*, and *yehD*) were significantly more prevalent than low-frequency virulence markers after Bonferroni correction (adjusted *P* < 0.05).

Many additional virulence factors occurred at low frequencies and collectively contributed to the overall heterogeneity in virulence-gene content, and all 20 isolates were predicted to be potentially pathogenic (Table 1).

### Genetic diversity of clinically relevant *E. coli* lineages

Core genome multilocus sequence typing (cgMLST; EnteroBase cgMLST v1) combined with HierCC clustering revealed substantial genetic diversity among the 20 *E. coli* isolates. A GrapeTree phylogeny constructed using the NINJA neighbor-joining algorithm revealed multiple sequence types (STs) and distinct clonal groupings (Fig. 3). HierCC analysis further indicated both closely related clusters and distantly related isolates, supporting the coexistence of diverse lineages within this system. Phylogroup classification further supported the observed genetic diversity, with isolates distributed across multiple phylogroups, including A, B1, B2, D, and E, as well as an ABD assignment (Fig. 3).

**Figure 3:**
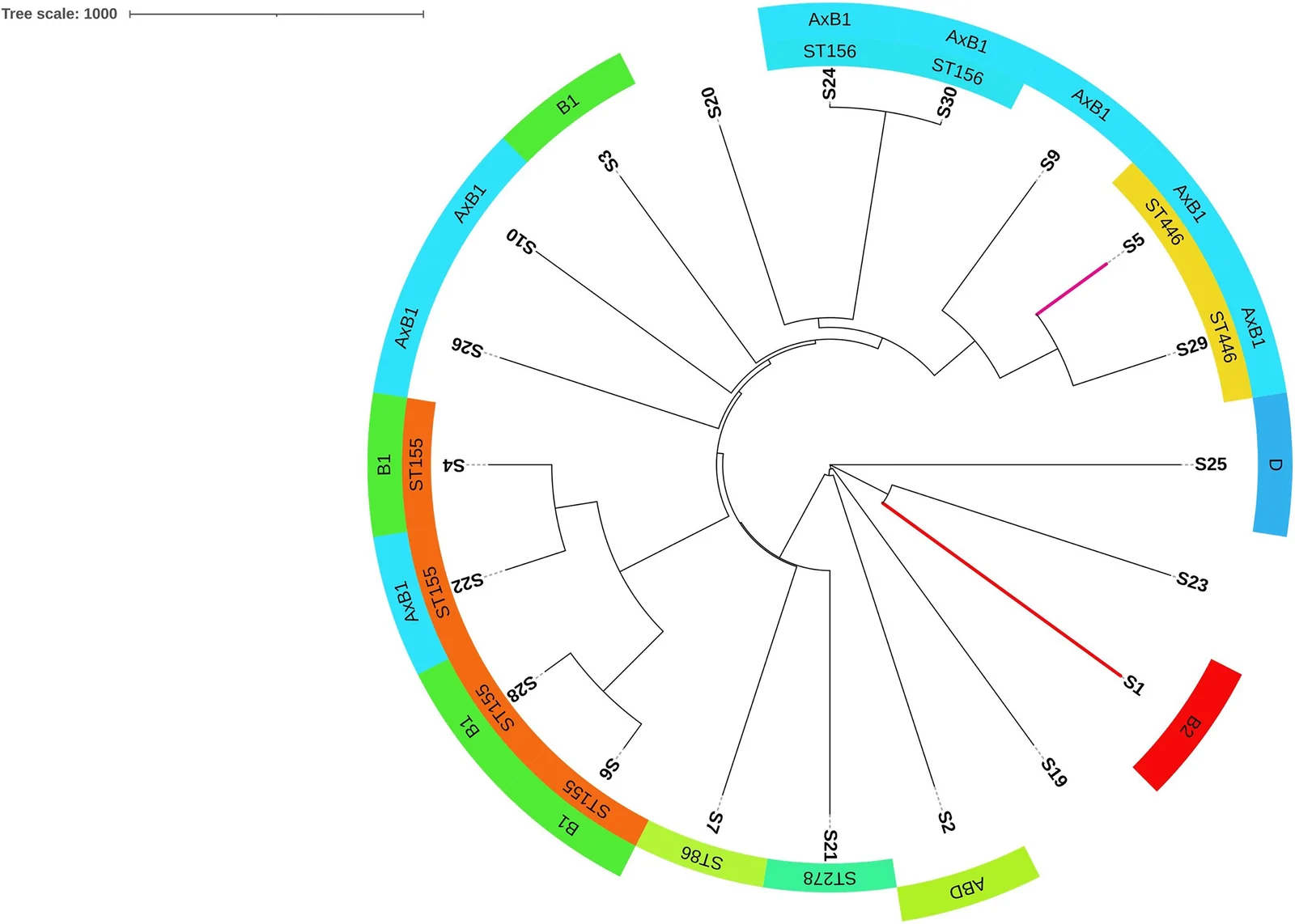
Phylogenetic tree illustrating the relationships among freshwater *E. coli* isolates (*n* = 20) from the Greys-Hoback watershed, inferred using EnteroBase version 1.2.0 cgMLST (cgMLST V1 + HierCC V1); based on NINJA NJ algorithm and visualized and annotated in Interactive Tree of Life (iTOL) version 7.2.2. The outer annotation rings indicate the sequence type (ST) and phylogroup assigned to each isolate.

Several STs belonged to recognized clonal complexes including the ST155 complex (S4, S6, S22, S28), ST156 complex (S24, S30), ST446 complex (S5, S29), and ST86 complex (S7). Other isolates were classified as singletons without an assigned complex, underscoring the presence of both common and rare lineages in this recreational system.

Notably, isolate S1 (ST646; serotype O4: H7; *fimH5*) carried an expanded ExPEC-associated virulence repertoire, including the yersiniabactin siderophore system (*fyuA, irp2*), group 2 capsule loci (*kpsE, kpsMII*), P fimbriae (*papA, papC*), the uropathogenic-specific protein gene *usp*, and the heme-acquisition gene *chuA*. Together, these findings suggest that this system harbors lineages with both enhanced virulence potential and a substantial mobile resistome.

Additionally, serotype prediction based on O- and H-antigen loci was performed using EnteroBase (Table 2), identifying a broad range of O: H combinations, including O4: H7, O7: H21, O8: H7, O17: H41, O23: H25, O25: H21, O38: H39, O84: H14, O85: H8, O87: H7, O98: H41, O134: H31, O153: H25, O27: H21, O156: H21, and O184: H48, as well as two isolates (S4 and S7) with only H-antigen assignments (H21 and H7, respectively).

**Table 2:** Genetic profiles of *E. coli* isolates (*n* = 20) from the Greys-Hoback watershed, including MLST sequence types, cgMLST allele numbers, O: H serotypes, and fimH alleles, highlighting genetic diversity.

Isolates	MLST	cgMLST	Serotype	FimTyper
S1	646	166 067	O4:H7	fimH5
S2	1087	89 388	O98:H41	-
S3	442	105 373	O156:H21	fimH32
S4	155	114 842	H21	fimH121
S5	1704	125 114	O87:H7	fimH31
S6	56	76 819	O25:H21	fimH54
S7	1308	4609	H7	fimH32
S9	196	196 780	O8:H7	fimH87
S10	937	55 523	O184:H48	fimH38
S19	6975	90 215	O38:H39	fimH30
S20	2144	43 895	O84:H14	-
S21	336	98 481	O27:H21	fimH297
S22	58	101 148	O7:H21	fimH32
S23	569	14 556	O134:H31	fimH5
S24	156	138 645	O23:H25	fimH38
S25	720	89 793	O17:H41	fimH65
S26	2522	132 463	O85:H8	fimH38
S28	56	104 712	O153:H25	fimH54
S29	666	4530	O8:H7	fimH31
S30	156	138 645	O23:H25	fimH38

### Plasmid-associated AMR in selected isolates

Contig-level analysis of isolates S5, S7, and S30 refined the genomic localization of AMR determinants and revealed distinct plasmid-associated patterns. In S5, multiple resistance genes, including *floR, tet(A), aph(6)-Id, aph(3′')-Ib, sul2*, and *bla*_TEM-150_, were localized to a plasmid cluster assigned by MOB-suite to an IncF-type plasmid with conjugative features. This finding supports the presence of an MDR plasmid in S5.

In S30, five resistance genes, *floR, tet(A), aph(6)-Id, aph(3′')-Ib*, and *sul2*, co-localized on a single plasmid-associated contig. A separate contig carrying *bla*_EC-18_ was classified as chromosomal, indicating that the β-lactamase gene was not part of the plasmid-associated MDR region. Although the S30 plasmid lacked identifiable mobility-associated features and was predicted to be non-mobilizable, it still represented a discrete plasmid-associated resistance element.

In contrast, S7 yielded conflicting results between analytical approaches. While plasmid replicon screening detected the presence of plasmid-associated sequences, MOB-suite classified the *tet(A)*-containing contig as chromosomal and identified a separate plasmid contig lacking AMR genes. Therefore, S7 was considered ambiguous and was not retained as a confirmed plasmid-associated AMR case.

## Discussion

The findings in this study support the concept that recreational freshwater environments can serve as reservoirs of potentially mobile MDR gene clusters. Only 20 of 218 confirmed isolates displayed clinically relevant resistance phenotypes, and six were classified as MDR. This classification followed established criteria for interpreting MDR (resistant to three or more antimicrobial classes) (Magiorakos *et al*. 2012). Nevertheless, these findings remain important because the watershed is actively used for recreation and lies within a broader One Health interface where human activity, wildlife, livestock, and surface water intersect. In this setting, *E. coli* serves primarily as an indicator of fecal contamination rather than a direct measure of pathogen risk. However, previous work has shown that indicator-positive waters can contain isolates with clinically relevant resistance and virulence traits (Franz *et al*. 2015, Korajkic *et al*. 2018). These results provide genomic evidence that even rural freshwater systems can serve as reservoirs for resistant *E. coli*, with potential relevance to human exposure.

Resistance in this watershed was dominated by tetracycline and streptomycin, with lower frequencies of chloramphenicol and ampicillin resistance and no phenotypic resistance to ceftriaxone or ceftiofur (Fig. 1). This pattern suggests that resistance within the watershed is primarily driven by widely distributed legacy, non-ESBL determinants of resistance rather than by high-risk ESBL phenotypes commonly associated with clinical settings. Similar patterns have been reported in aquatic environments, where tetracycline resistance is often the predominant phenotype, especially in systems influenced by mixed anthropogenic and animal inputs (Haberecht *et al*. 2019, Kaur *et al*. 2026). At the watershed scale, this pattern is consistent with the integration of inputs from agricultural runoff, wildlife, and human-associated sources, allowing resistance determinants from different origins to co-occur (Kraemer *et al*. 2022).

WGS provided important context for interpreting these patterns. Although *bla*_EC_ variants were detected across the sequenced isolates, these findings should be interpreted alongside the observed susceptibility to third-generation cephalosporins and the absence of common ESBL genes (Fig. 2a). Notably, phenotypic resistance exceeded the detected genotypic determinants for several agents: 16 of 20 isolates were tetracycline-resistant but only 10 carried a *tet* gene, and 10 were streptomycin-resistant despite only 5 carrying *aph(3′')-Ib*/*aph(6)-Id*, suggesting contributions from efflux, chromosomal mutations, and more (Darby *et al*. 2023). Together, these findings highlight the importance of integrating phenotypic and genomic data for accurate interpretation.

Overall, these genomes do not indicate a strong dominance of clinically critical cephalosporin resistance. Instead, the consistent signal was the repeated detection of non-β-lactam resistance genes, including *tet, sul2, aph(3′')-Ib, aph(6)-Id*, and *floR*. The co-occurrence, particularly in MDR isolates, indicates a mobile resistome consistent with environmental and animal-associated *E. coli* populations (Fig. 2a). These findings support the idea that recreational waters may function not only as reservoirs of resistant bacteria but also as environments where resistance genes persist and may exchange.

To better understand the genomic context of resistance, plasmid-associated elements were analyzed in detail. Most isolates carried plasmid replicons, and IncFIB and IncFII were the dominant backbones (Fig. 2b). IncF plasmids are widely recognized as important vehicles for the persistence and dissemination of AMR and virulence traits in *E. coli* and related Enterobacterales (Pitout and Chen 2023). Their presence does not demonstrate active horizontal transfer within the watershed; however, they indicate that many isolates harbor genomic architectures that are compatible with the maintenance and spread of adaptive traits. The presence of multi-replicon profiles and the clustering of multiple resistance genes in isolates such as S5, S24, and S30 further support the presence of a mobile gene pool within this system.

The contig-level analysis suggests that plasmids contribute substantially to resistance gene carriage in this collection, though its contribution varied across isolates. In isolate S5, the co-localization of *floR, tet(A), aph(6)-Id, aph(3′')-Ib, sul2*, and *bla*_TEM-150_ genes on a conjugative IncF-type plasmid is of certain concern, since physical linkage of these determinants on a mobile element selects for co-selection and horizontal transfer; exposure to any one agent could sustain the entire cassette. IncF plasmids are well-recognized vehicles for MDR in *Enterobacteriaceae* (Stein et al. 2024), and our findings are consistent with this role. The recurrence of the same *floR, tet(A), aph(6)-Id, aph(3′')-Ib, sul2* genes in S30 potentially indicates a conserved resistance region circulating among these isolates. However, it remained limited to a plasmid contig while the intrinsic *bla*_EC-18_ stayed chromosomal. This partial segregation usefully distinguishes mobile, potentially transferable resistance from determinants that may be tied to the chromosome and therefore less likely to disseminate. On the contrary, isolate S7 was harder to decipher as plasmid replicons were detected, yet its AMR genes assembled onto chromosomal contigs, leaving the association unresolved. We consider this most likely an artifact of assembly fragmentation rather than a true absence of plasmid-borne resistance, a recognized limitation of reconstructing repetitive plasmid sequences from short reads, as described previously (Arredondo-Alonso *et al*. 2017, Teixeira *et al*. 2025).

These findings highlight both the contribution of plasmids to resistance gene clustering and the limitations of plasmid reconstruction using short-read sequencing approaches (Arredondo-Alonso *et al*. 2017). Importantly, the detection of plasmid-associated MDR regions in isolates from recreational waters underscores the potential for environmental dissemination of clinically relevant resistance determinants beyond traditional clinical or agricultural settings.

In addition to AMR, all sequenced isolates carried multiple virulence-associated genes, and several encoded combinations consistent with both extraintestinal and enterotoxigenic potential. This aligns with previous studies showing that environmental *E. coli* can harbor overlapping resistance and virulence traits, including in surface water and wastewater-impacted systems (Franz *et al*. 2015, Gomes *et al*. 2024). However, these findings still need to be interpreted with caution, as genome-based pathogenicity predictions and virulence gene profiles reflect potential rather than confirmed clinical behavior, as they do not account for gene expression, infective dose, or host susceptibility. Accordingly, these predictions should be considered indicative rather than definitive measures of pathogenic risk.

The serotypic heterogeneity observed here is consistent with repeated introduction of resistant *E. coli* from multiple fecal inputs rather than the expansion of a single dominant clone. Broad serotype and lineage diversity is a recurrent feature of recreational and mixed-use surface waters, where *E. coli* populations commonly include numerous co-occurring serotypes and phylogenetic backgrounds and, in some settings, substantial MDR burdens (Nadya *et al*. 2016, Haberecht *et al*. 2019). Because O: H antigen loci can occur on different chromosomal backgrounds, serotype data alone do not establish source attribution; however, when interpreted together with MLST/cgMLST, such heterogeneity provides complementary evidence of population-level diversity and repeated inputs rather than recent clonal expansion (Ingle *et al*. 2016). This interpretation is further supported by aquatic AMR studies showing that resistant *E. coli* are phylogenetically dispersed and carry diverse resistome across water systems, again arguing against a single dominant resistant lineage (Haberecht *et al*. 2019, Ogura *et al*. 2020).

Phylogenetic analyses further demonstrated that the resistant *E. coli* population in this watershed is highly diverse (Fig. 3). MLST, cgMLST, serotyping, and *fimH* typing revealed multiple lineages, including both clustered and genetically distinct isolates, consistent with repeated environmental introduction rather than clonal expansion. The distribution of AMR isolates across multiple *E. coli* phylogroups further supports the presence of a genetically heterogeneous population within the watershed and is consistent with potential contributions from multiple ecological sources. Several isolates belonged to recognized clonal complexes, including ST155, ST156, ST446, and ST86, while others were singletons, reflecting a mix of common and rare lineages. This pattern is consistent with the ecology of mixed-use watersheds, where bacterial inputs come from wildlife, livestock, septic systems, and human activity. Similar heterogeneity has been reported in other environmental water studies, including systems influenced by wastewater and landscape-scale runoff (Haberecht *et al*. 2019, Kaur *et al*. 2026). Accordingly, lineage diversity may be as informative as overall resistance prevalence, indicating a distributed and continuously replenished AMR burden.

Several limitations should be considered when interpreting these findings. First, genomic analyses were limited to 20 phenotypically resistant isolates and therefore do not represent the full *E. coli* population present in the watershed. Second, the study design does not allow direct source attribution; incorporating microbial source tracking or land-use data would strengthen ecological interpretation. Third, plasmid reconstruction from short-read assemblies remains challenging, and detection of plasmid replicons does not confirm plasmid completeness or transferability. Finally, pathogenicity predictions should be interpreted as screening-level evidence rather than definitive indicators of human health risk.

Despite these limitations, this study provides a framework for linking routine water-quality monitoring with genomic surveillance of AMR. Incorporating AST, targeted WGS, and plasmid analysis into recreational water monitoring could improve early detection of high-risk resistance profiles and support overall risk assessment. More broadly, these findings support the inclusion of recreational freshwater systems in One Health AMR surveillance efforts, particularly in regions where environmental, agricultural, and human factors intersect.

Collectively, these findings highlight recreational waters as under-recognized reservoirs of potentially mobile MDR gene clusters with potential zoonotic relevance, emphasizing their importance within One Health AMR surveillance frameworks.

## Data Availability

Whole-genome sequencing data have been deposited in NCBI under BioProject PRJNA1146023. Accession numbers as listed in Table 1.
